# Noonan Syndrome and Heart Failure in a Postpartum Patient

**DOI:** 10.7759/cureus.82109

**Published:** 2025-04-11

**Authors:** Jose Alberto Domínguez-López, Davina Desireé Teco-Mendoza, Luis E Mendoza-Razo

**Affiliations:** 1 Genetics, Universidad Autónoma de Chiapas, Instituto de Salud del Estado de Chiapas, Tuxtla Gutiérrez, MEX; 2 General Medicine, Instituto de Salud de Chiapas, Tuxtla Gutiérrez, MEX; 3 Intensive Care Unit, Instituto de Salud de Chiapas, Tuxtla Gutiérrez, MEX

**Keywords:** critical care cardiology, critical care in obstetrics, heart disease in pregnancy, management of heart failure, noonan syndrome

## Abstract

A 29-year-old postpartum woman with no prior Noonan syndrome diagnosis was admitted for recurrent chest pain, dyspnea, and palpitations. She had two adverse pregnancy outcomes, including a neonatal death due to perinatal asphyxia. Physical examination revealed characteristic facial features, and echocardiography confirmed biventricular failure, hypertrophic cardiomyopathy, and a 2.5 cm intracavitary thrombus in the left ventricle. She was treated with heart failure management, anticoagulation, and diuretics before being referred to a specialized cardiology center. This case highlights the challenges in diagnosing and managing cardiovascular complications in suspected Noonan syndrome.

## Introduction

Noonan syndrome, first described in 1968, is characterized by short stature, hypertelorism, webbed neck, chest deformity, and congenital heart defects [1]. In Mexico, its incidence is estimated to be between one in 1,000 and one in 2,500 newborns, similar to the incidence in other countries [2]. Mutations in genes encoding proteins in the Ras-mitogen-activated protein kinase (MAPK) signaling pathway cause Noonan syndrome and other related disorders, such as Noonan syndrome with multiple lentigines (formerly known as LEOPARD (lentigines, electrocardiographic conduction abnormalities, ocular hypertelorism, pulmonary stenosis, abnormal genitalia, retarded growth, and deafness) syndrome), cardiofaciocutaneous syndrome, and Costello syndrome. These disorders share a common genetic origin and are grouped under the term "rasopathies." Despite advances in genetics, about 20% of patients do not have an identified genetic cause, so diagnosis remains based on clinical evaluation [3]. This syndrome exhibits significant clinical and genetic heterogeneity. The primary cardiac defects include pulmonary valve stenosis and hypertrophic cardiomyopathy. Less commonly reported defects include ventricular septal defect, patent ductus arteriosus, tetralogy of Fallot, aortic coarctation, and mitral stenosis [1,3].

## Case presentation

A 29-year-old woman in the postpartum period, originally from San Cristóbal de Las Casas, Chiapas, was admitted to the "Dr. Jesús Gilberto Gómez Maza" General Hospital. She reported recurrent episodes of chest pain, shortness of breath, and palpitations that subsided with rest. Of note, her parents are consanguineous. She had no prior history of genetic diagnoses or evaluations.

Three days before her admission to the emergency department, she was at the community maternal and neonatal hospital due to labor at 38 weeks of gestation. Unfortunately, the newborn died a few minutes after birth due to prolonged labor and neonatal asphyxia. This was her second pregnancy; the first resulted in a miscarriage at 18 weeks of gestation. While at the maternal and neonatal hospital, she was also treated for pneumonia, which began with shortness of breath, yellowish sputum, and tachypnea. Rapid tests for COVID-19 and influenza were negative. She was discharged with an improvement in her pneumonia symptoms, although the causal agent was not identified, and she was referred for suspected heart failure management.

**Figure 1:**
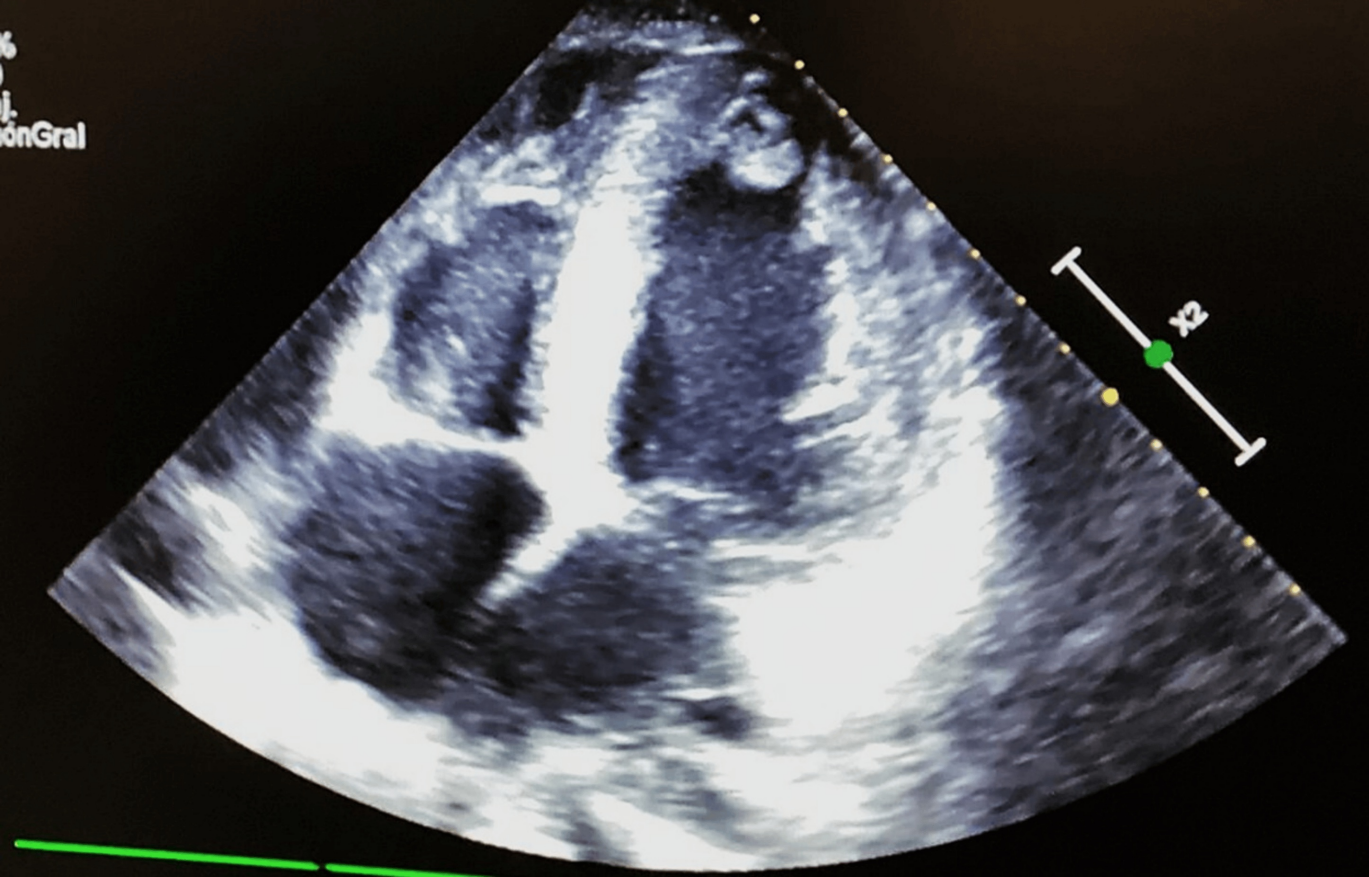
Echocardiogram Biventricular failure with a left ventricular ejection fraction (LVEF) of 33%, concentric hypertrophy without signs of pulmonary hypertension, and systolic and diastolic dysfunction of both ventricles. An intracavitary thrombus was observed in the left ventricle, measuring 2.5 × 2 cm, along with dilation of both the right and left chambers. The right atrial systolic pressure (RASP) was 3 mmHg.

**Figure 2:**
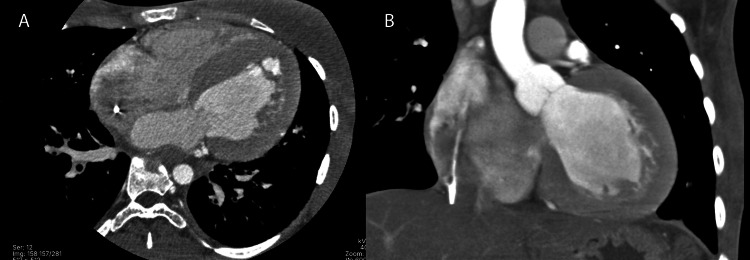
Contrast-enhanced thoracic CT angiography A) Sagittal view shows concentric left ventricular hypertrophy with septal predominance. B) Axial view reveals moderate dilation of the right atrium and the pulmonary artery trunk. Additionally, a hyperdense intracavitary mass is identified, located in the right atrium and adjacent to the endocardial wall.

Upon admission, her vital signs were as follows: blood pressure - 90/60 mm Hg, mean arterial pressure (MAP) - 70 mmHg, heart rate: 70 bpm, respiratory rate - 20 breaths per minute, and oxygen saturation - 86%. During the physical examination, the patient was conscious, alert, and responsive to external stimuli, with a Glasgow Coma Scale score of 15. There were no signs of meningeal irritation, and her deep tendon reflexes were intact. The clinical phenotype included a triangular face with hypertelorism, low-set ears, and a short, broad neck. Her chest was short with symmetrical expansion. Auscultation revealed bilateral parahilar crackles and decreased breath sounds at both lung bases. There was a holosystolic murmur at the mitral and tricuspid areas, along with the presence of an S3 gallop. The electrocardiogram showed ventricular extrasystoles and an arrhythmic pattern. In the intensive care unit (ICU), an echocardiogram was performed (Figure 1 and Figure 2).

Treatment for heart failure was initiated with sacubitril/valsartan, digoxin, spironolactone, and furosemide. Due to the presence of the intracavitary thrombus, anticoagulation was started with acenocoumarol, followed by enoxaparin, achieving an INR of 3.01. The patient experienced mild thrombocytopenia and mild anemia (Table 1). Additionally, the patient presented with acute kidney injury (Kidney Disease: Improving Global Outcomes (KDIGO) II), with a urea level of 96 mg/dL and creatinine of 0.9 mg/dL, and urine output of 0.4 mL/kg/hour over 12 hours. Due to the need for specialized management of the intracavitary thrombus, the patient was referred to a specialized cardiology center for continued treatment.

**Table 1:** Laboratory results

Laboratory test	Result	Reference values
Hemoglobin	10.4 g/dL	12.0-16.0 g/dL
Platelets	97,000/µL	150,000-450,000/µL
Leukocytes	7,400/µL	4,000-11,000/µL
Urea	96 mg/dL	10-50 mg/dL
Creatinine	0.9 mg/dL	0.6-1.3 mg/dL
Total bilirubin	0.9 mg/dL	0.1-1.2 mg/dL
Aspartate aminotransferase	130 U/L	8-40 U/L
Alanine aminotransferase	127 U/L	7-56 U/L
Sodium	125 mEq/L	135-145 mEq/L
Potassium	3.7 mEq/L	3.5-5.0 mEq/L
Chloride	88 mEq/L	96-106 mEq/L
Phosphorus	4.1 mg/dL	2.5-4.5 mg/dL
Procalcitonin	4 ng/mL	<0.5 ng/mL

## Discussion

We describe the case of a young patient without a prior diagnosis of Noonan syndrome who was admitted during the immediate postpartum period. She was diagnosed with and treated for hypertrophic cardiomyopathy, an intracavitary thrombus, and community-acquired pneumonia. Congenital heart defects in patients with Noonan syndrome are determined by hemodynamic alterations, with no direct repercussions linked solely to the syndrome itself. Despite advances in genetics, about 20% of patients do not have an identified genetic cause, so diagnosis remains based on clinical evaluation [3]. This syndrome exhibits significant clinical and genetic heterogeneity. The primary cardiac defects include pulmonary valve stenosis and hypertrophic cardiomyopathy. Less commonly reported defects include ventricular septal defect, patent ductus arteriosus, tetralogy of Fallot, aortic coarctation, and mitral stenosis [1,3].

However, it is important to understand the high prevalence of pulmonary valve stenosis (affecting up to 40% of patients), atrial septal defects, and hypertrophic cardiomyopathy (seen in up to 30% of patients), among other cardiac conditions [3, 4]. In our patient, the presence of a 2.5 cm intracavitary thrombus and hypertrophic cardiomyopathy was evident. A retrospective study conducted at Yale-New Haven Hospital from 2012 to 2020, involving patients diagnosed with Noonan syndrome and a total of 10 pregnancies, found that the majority (n = 8) had experienced heart disease [5]. No complications were observed during pregnancy or in the long-term postpartum period, except for one patient who had a pregnancy ending in fetal death at five weeks of gestation. In the case presented, both pregnancies in the patient resulted in miscarriage or fetal death, highlighting the importance of studying patients with Noonan syndrome in hospitals and regions where conditions are not ideal. The main factor for complications in these cases is the lack of prenatal care and timely attention [6,7].

## Conclusions

Currently, a wide variety of genes associated with Noonan syndrome are known, although genetic panels or tests are not indicated, as detection relies on clinical evaluation. Our patient received genetic counseling from an institutional geneticist, but no genetic test results were available at the time of this study. This case underscores the complexity of managing cardiovascular diseases in patients suspected of having Noonan syndrome and the need for specialized care in ICUs.
